# Chronic exposure of diesel exhaust particles induces alveolar enlargement in mice

**DOI:** 10.1186/s12931-015-0172-z

**Published:** 2015-02-07

**Authors:** Kelly Yoshizaki, Jôse Mára Brito, Henrique T Moriya, Alessandra C Toledo, Sandra Ferzilan, Ana Paula Ligeiro de Oliveira, Isabel D Machado, Sandra HP Farsky, Luiz FF Silva, Milton A Martins, Paulo HN Saldiva, Thais Mauad, Mariangela Macchione

**Affiliations:** Department of Pathology, Experimental Air Pollution Laboratory, LIM05 - School of Medicine, University of Sao Paulo, Av. Dr. Arnaldo 455, 1 andar, sala 1150, Cerqueira César, São Paulo, SP CEP 01246-903 Brazil; Biomedical Engineering Laboratory, Escola Politecnica, University of Sao Paulo, Sao Paulo, Brazil; Department of Medicine, School of Medicine, University of Sao Paulo, Sao Paulo, Brazil; Department of Clinical and Toxicological Analysis, School of Pharmaceutical Sciences, University of Sao Paulo, Sao Paulo, Brazil; Pos Graduate Program in Biophotonics Applied to Health Sciences, Nove de Julho University, Sao Paulo, Brazil

**Keywords:** Air pollution, Diesel exhaust particulate, Lung, Lymphocyte, Mice

## Abstract

**Background:**

Diesel exhaust particles (DEPs) are deposited into the respiratory tract and are thought to be a risk factor for the development of diseases of the respiratory system. In healthy individuals, the timing and mechanisms of respiratory tract injuries caused by chronic exposure to air pollution remain to be clarified.

**Methods:**

We evaluated the effects of chronic exposure to DEP at doses below those found in a typical bus corridor in Sao Paulo (150 μg/m^3^). Male BALB/c mice were divided into mice receiving a nasal instillation: saline (saline; n = 30) and 30 μg/10 μL of DEP (DEP; n = 30). Nasal instillations were performed five days a week, over a period of 90 days. Bronchoalveolar lavage (BAL) was performed, and the concentrations of interleukin (IL)-4, IL-10, IL-13 and interferon-gamma (INF-γ) were determined by ELISA-immunoassay. Assessment of respiratory mechanics was performed. The gene expression of Muc5ac in lung was evaluated by RT-PCR. The presence of IL-13, MAC2+ macrophages, CD3+, CD4+, CD8+ T cells and CD20+ B cells in tissues was analysed by immunohistochemistry. Bronchial thickness and the collagen/elastic fibers density were evaluated by morphometry. We measured the mean linear intercept (Lm), a measure of alveolar distension, and the mean airspace diameter (D0) and statistical distribution (D2).

**Results:**

DEP decreased IFN-γ levels in BAL (p = 0.03), but did not significantly alter IL-4, IL-10 and IL-13 levels. MAC2+ macrophage, CD4+ T cell and CD20+ B cell numbers were not altered; however, numbers of CD3+ T cells (p ≤ 0.001) and CD8+ T cells (p ≤ 0.001) increased in the parenchyma. Although IL-13 (p = 0.008) expression decreased in the bronchiolar epithelium, Muc5ac gene expression was not altered in the lung of DEP-exposed animals. Although respiratory mechanics, elastic and collagen density were not modified, the mean linear intercept (Lm) was increased in the DEP-exposed animals (p ≤ 0.001), and the index D2 was statistically different (p = 0.038) from the control animals.

**Conclusion:**

Our data suggest that nasal instillation of low doses of DEP over a period of 90 days results in alveolar enlargement in the pulmonary parenchyma of healthy mice.

**Electronic supplementary material:**

The online version of this article (doi:10.1186/s12931-015-0172-z) contains supplementary material, which is available to authorized users.

## Introduction

Traffic is a major contributor to air pollution in cities, and traffic-related exposure has been shown to induce acute inflammation in humans, both in chamber studies using diesel exhaust [1] and in “real-life” environments such as road tunnels [2]. In urban centres, diesel exhaust particles (DEP) are considered to be the hazardous pollutants released from automotive engines due to their aerodynamic and chemical characteristics [3,4].

Smoking has been considered the most important risk factor for chronic obstructive pulmonary disease (COPD) [5]. However, many COPD cases occur in non-smokers. Indeed, it is estimated that 25-45% of the patients with COPD worldwide have never smoked [6]. Nonsmoking-related risk factors such as genetic syndromes (α1-antitrypsin deficiency) as well as occupational exposures (outdoor pollution, second-hand smoke, biomass smoke), chronic asthma and tuberculosis also contribute to the development and progression of COPD [5-7].

Long term exposure to air pollution induces chronic inflammatory changes in the airways [8] and increases the risk of airway obstruction [9]. It is not known whether chronic exposure to air pollution leads to emphysematous changes in the lungs. Lopes et al. [10] that showed that particles emitted by traffic can worsen the development of emphysema in mice treated with papain, but hitherto it has not been demonstrated that air pollution alone could lead to structural alterations in the airspaces of the lungs.

In previous work from our laboratory, Yoshizaki et al. [11] showed that instillation of 30 μg/10 μl of DEP over a period of 60 days induced respiratory tract inflammation and increased mucus content in the nose. A pilot study using these samples showed a time dependent DEP-induced alveolar enlargement, reaching statistical significance at 60 days (data not shown).

In this present study, we hypothesised that chronic exposure of mice to DEP would result in significant airway and lung parenchymal inflammation and changes in the alveolar structure. Therefore, we investigated the effect of a lower dose of DEP for a longer exposure period (90 days) in mice, focusing on the development and pathophysiological consequences of the inflammatory response. We evaluated bronchial epithelium thickness, IL-13 expression and Muc5ac RNA expression in pulmonary tissue. We also determined the number of inflammatory cells and cytokine expression [interleukin (IL)-4, IL-10, IL-13 and interferon-gamma (INF-γ)] in the BAL. We further quantified alveolar diameter (mean linear intercepts), elastic and collagen and the number of MAC2+ macrophages, CD20+ B cells, CD3+, CD4+ and CD8+ T cells in lung parenchyma.

## Materials and methods

### Ethics statement

This study was approved by the Ethics Committee for Research of the São Paulo University Medical School (CAPPesq-FMUSP) number 0571/08.

### Particle collection and particle composition analysis

Diesel particles were collected after 1 day of routine operation of a bus from São Paulo city’s metropolitan fleet that was equipped with a Mercedes Benz MB1620, 210-hp engine with a Euro III emission profile, which lacked an electronic fuel injector. The PM was obtained. The diesel fuel used in Sao Paulo contains 500 ppm of sulfur. The diesel particulate material was collected with a particle retainer that is being tested on diesel vehicles to reduce PM emissions and was stored at 4°C for further toxicological and analytical studies. The particle retainer consists of a bimetallic filter that creates a field capable of retaining the PM emitted from the exhaust of diesel buses. As previously published, the characteristics of the DEPs were analyzed according to (a) concentrations of elements, which were determined by energy-dispersive X-ray fluorescence spectrometry (mean ± SEM) (ppb): Ni (181 ± 37), S (626 ± 416), V (37 ± 13), Pb (50 ± 47), Fe (74,556 ± 2,266), Cd (29 ± 8), Cr (161 ± 116) and Cu (17 ± 1); and (b) concentrations of polycyclic aromatic hydrocarbons (PAHs), determined by high-performance liquid chromatograph (ng/g): Naphthalene (49.23), Acenaphthylene (179.48), Fluorene (683.94), Anthracene (94.73), Pyrene (12,838.27), Benz[a]anthracene (1,162.73), Benzo[b]fluoranthene (789.93), Benzo[k]fluoranthene (562.28) and Benzo[a]pyrene (1,642.28) [11]. The distribution of particle sizes, as measured by their volume and surface, and the diameters encompassing 90%, 50%, and 10% of the particulate matter were determined by laser diffraction (Long Bench Mastersizer, Malvern Instruments, Malvern, UK). The analysis was performed at the Laboratory of Technological Characterization, Department of Mining and Petroleum Engineering, Polytechnic School, University of São Paulo, São Paulo, Brazil.

The DEP was resuspended in saline at 10 mg/mL for 2 h through magnetic stirring and was sonicated for 30 min. Next, the DEP was diluted to 30 μg of DEP in 10 μL of saline and stored at −20°C until use.

### Exposure protocol

Sixty BALB/c 8-week-old male mice (body weight ca. 20–25 g) were assigned to two groups: (a) Saline (n = 30): animals received an intranasal instillation of 10 μL saline solution (0.9% NaCl); and (b) DEP (n = 30): intranasal instillation of 30 μg DEP/ 10 μL of saline (5 μl in each nostril). This protocol was conducted for 90 days, five days a week [11,12].

All animals received humane care in compliance with the “Principles of Laboratory Animal Care” formulated by the National Society for Medical Research and the “Guiding Principles in the Care and Use of Animals” approved by the Council of the American Physiological Society. Our Institutional Animal Care and Use Committee approved all protocols in this study.

### Respiratory mechanics

After 90 days of intranasal instillation, animals were anaesthetised with sodium pentobarbital (50 mg/kg body weight, intraperitoneally), tracheotomised and connected to a small animal ventilator (FlexiVent, Scireq, Montreal, Canada). The animals were paralysed with pancuronium bromide (0.2 mg/kg body weight, intraperitoneally). The forced oscillation technique was applied in basal conditions with the constant phase model characterised by the following parameters: airway resistance (Raw), tissue damping (Gtis) and tissue elastance (Htis). Next, a methacholine (25 mg/mL) challenge was performed. Total respiratory system resistance (R) was evaluated using the single compartment model in basal condition and after the methacholine challenge. After respiratory mechanics assessment, animals were sacrificed by exsanguination [11].

### Bronchoalveolar lavage

Bronchoalveolar lavage (BAL) of lungs was performed on half of the mice from each study group. Immediately after respiratory mechanics assessment, BAL was performed by introducing 0.5 mL sterile phosphate-buffered saline (PBS) into the lungs via a tracheal cannula, and the recovered fluid was kept in a test tube on ice. This procedure was repeated three times. The fluid collected was centrifuged at 1810 rpm for 10 minutes at 5°C to separate cells from the supernatant. The supernatant was stored at −70°C and subsequently used for ELISA analysis. The cell pellet was resuspended in 300 μl PBS. A volume of 100 μl of resuspended pellet was removed and stored in an Eppendorf tube with 400 μl PBS. Total cells were counted using an improved Neubauer hemocytometer chamber and an optical microscope with a 400X zoom. BAL differential cell counts were performed on cytocentrifuge slides prepared by the centrifugation of each sample (100 μl BAL) at 450 rpm for 6 minutes (Cytospin 2, Shandon Scientific, Pittsburgh, PA, USA). These slides were stained using Diff-Quick (Romanowsky) stain (Muto Kagaku Co., Tokyo, Japan), and differential counts of at least 300 cells were made according to standard morphologic criteria. Macrophages, lymphocytes, eosinophils, and neutrophils were enumerated [13].

### Measurement of IL-4, IL-10, IL-13 and INF-γ

The levels of IL-4, IL-10, IL-13 and INF-γ were quantified in BAL cell lysates using ELISA kits purchased from R&D Systems (Minneapolis, MN, USA). ELISA was performed in accordance with the manufacturer’s instructions. IL-4, IL-10, IL-13 and INF-γ levels were obtained using standard curves. Each sample was assayed in triplicate [14].

### Histological analysis

Histological analysis of lungs was performed on the remaining half of the mice from each study group. After anaesthesia and exsanguinations of the animals, the lungs were immediately inflated with warmed 1% low melt agarose at 25–30 cmH_2_O. Pressure was maintained for approximately 1 minute, by which time the agarose began to harden substantially. The trachea was then tied with a line, and the whole animal was placed in a refrigerator at 4°C for at least 2 h [15]. The chest was opened, and the lungs were then removed from the chest (10 lungs per group). Longitudinal sections were fixed in 4% neutral buffered formalin. Tissues were subsequently embedded in paraffin, sectioned 5-μm-thick, and stained with hematoxylin and eosin (H&E) to evaluate general morphology [11].

### Quantification of bronchial epithelial thickness

For each animal, five transversally cut bronchioles with an adequate cross sectional profile (less than 10% of variation in maximal and minimal diameter) were analysed. Using a Leica DMR microscope attached to both a JVC TK-C 1380 colour video camera and an image analysis software system (Image Pro-Plus 4.1, Media Cybernetics, Silver Spring, Md., USA), we digitalised the microscopic images in a high-resolution video and viewed them through an eyepiece with a known area. The average epithelium thickness was determined by measuring the basal membrane limit and the apical membrane limit (magnification of 1380X) [11]. Values measured for each of the five airways were averaged to provide a single data point for each animal.

### Mean linear intercept (Lm) and airspace enlargement

Lm was measured in airspaces adjacent to the pleura (distal) [16], and an algorithm was applied to perform quantitative characterisation of airspace enlargement [17].

Lm, an indicator of mean alveolar distension [18], was assessed in 10 non-overlapping fields of lung parenchyma per animal at × 400 magnification in digitised images [11].

The Lm can be used to estimate the surface area for gas exchange in the lung. Lm is a reliable index for a relatively homogeneous enlargement of airspaces. However, in the presence of spatial heterogeneities with large variability of airspace sizes, Lm did not significantly increase and sometimes even decreased compared with its value in normal tissue [17]. An automated method for measuring area and computed an equivalent diameter of each individual airspace that is independent of shape was developed [17]. We applied this automated method to cross section images obtained from the same microscope used to calculate Lm. Mean airspace diameter (D0) and an index based on the statistical distribution of D0 (D2) were calculated. This index is able to identify abnormal airspace enlargement under heterogeneous conditions because it gives more weight to the enlarged airspaces than the smaller ones. In this study, we calculated the Lm and the D2 to avoid bias due to spatial heterogeneity with large variability of airspace sizes.

### Immunohistochemistry

Five-μm thick sections were used to identify cells expressing IL-13, MAC2 + macrophages, CD3 + T cells, CD4 + T cells, CD8T+ cells and CD20 + B cells by immunohistochemistry. Briefly, sections were deparaffinised, and a 0.5% peroxidase in methanol solution was applied for 5 minutes to inhibit endogenous peroxidase activity. Antigen retrieval was performed with citrate solution for 20 minutes. Sections were incubated overnight with anti–IL-13 (1:60), anti-MAC2 (1:40.000), anti-CD3 (1:300), anti-CD4 (1:2000), anti-CD20 (1:20.0000) and anti CD8 (1:50) (Santa Cruz Biotechnology Inc., Santa Cruz, CA, USA). 3,3 diaminobenzidine (Sigma Chemical Co., St Louis, MO, USA) was used as a chromogen. The sections were counterstained with Harris haematoxylin (Merck, Darmstadt, Germany). All primary and secondary antibodies were applied to negative and positive controls. The slides were coded, and the researcher who performed the morphometrical analyses was blinded to the study groups. The expression of IL-13 was determined using digital image analysis, and a positive threshold was applied to all images. For each image, we measured the positively stained area within the epithelial layer and the epithelial area. The results are presented as percentages of positivity (proportion of positive area).

We also counted the number of CD3+, CD4+, CD8+, CD20+ and MAC2+ cells in ten parenchymal areas per animal. The inflammatory cells were manually counted and expressed as the number of positive cells within the epithelial layer per epithelial area. Briefly, we manually counted the number of positive cells in ten high power fields. In addition, using a 100 point grid of known area, we determined the corresponding proportion of air spaces and tissue in the same image. The results are presented as the number of cells per tissue area (cells/mm^2^).

### Collagen and elastic fibers analyses

Sections stained with Sirius red (for collagen fibers) and resorcin-fuchsin (for elastic fibers) were evaluated in the parenchyma region. We photographed 10 parenchyma fields per slide. The proportion of the area occupied by each type of fiber divided by tissue area was calculated for comparisons among groups.

### Muc5ac gene expression in lung tissue

#### RNA isolation

RNA was isolated from the lungs of seven animals of each group. Lungs were removed and immediately immersed in 2 mL of the Trizol Reagent (Invitrogen Life Technologies). Total RNA isolation and extraction were performed according to the manufacturer’s guidelines, as modified by Chomczynski and Sacchi [19], as previously described Yoshizaki et al. [11].

Briefly, each sample was quickly homogenised in Politron (Kinematic) and transferred to Eppendorf tubes. Samples were homogenised for five minutes at room temperature to allow the complete dissociation of nucleoprotein complexes. Next, we added 200 μL of chloroform (Merck), and the tubes were mixed thoroughly by inversion. The tubes were incubated at room temperature for two minutes, followed by centrifugation for 15 minutes at 12000 g and 4°C. After transferring the aqueous phase to a fresh tube, 500 μL of isopropyl alcohol (Merck) was added to precipitate the RNA and incubated for one hour at room temperature. Then, the tubes were centrifuged at 12000 g for 10 minutes at 4°C. The RNA pellets were washed with 75% ethanol (Merck) and centrifuged at 7500 g for five minutes at 4°C. The samples were then dissolved in DEPC water (water treated with RNase inhibitor, Diethylpyrocarbonate; Merck) and stored for 10 minutes at 60°C for complete dissolution of the RNA. The RNA concentration and purity were determined by measuring the absorbance at 260 and 280 nm.

#### Reverse transcriptase/polymerase chain reaction-real time

Reverse transcription of RNA to cDNA was performed using the following reaction mixture: 10 μg total RNA from each sample in 2 μL DEPC-treated water, 2 μL Oligo (dT) at 500 μg/mL (Invitrogen), 2 μL dNTP mix at 10 mM (Invitrogen), 8 μL 5x *first-strand buffer* (Invitrogen)*,* 2 μL DTT at 0.1 M, 2 μL RNaseOUT (Recombinant RNase Inhibitor, Invitrogen), and 2 μL *Superscript III RT* enzyme (200 U/μL). The reaction mixture was incubated at 50°C for 50 minutes, then at 70°C for 15 min, and finally stored at −20°C. Polymerase chain reaction was performed using *Rotor-Gene RG3000 (Corbett Research)* in a 20 μl reaction mixture containing: 1.5 U *Platinum®* Taq DNA polymerase (Invitrogen); 200 μM of each dNTP, 1.5X SYBR *Green*, 5% DMSO (Dimetil sulphoxide), 0.3 μM sense and anti-sense oligonucleotides to Muc5ac, and β-actin; 1.5 mM MgCl_2_; and 100 ηg cDNA. The level of each mRNA expression was normalised in relation to β-actin. The sequences used were Muc5ac sense 5′-ACGACACTTTTCAGTACCAATGAC-3′ and anti-sense 5′-GCTTCCTTACAGATGCAGTCCT-3′, as well as β-actin sense 5′CTGTGGCATCCACGAAACTA-3′ and anti-sense 5′-AGTACTTGCGCTCAGGAGGA-3′. The primer sets have been published by Lankford et al. [20] and were previously described by Yoshizaki et al. [11].

### Statistical analysis

Data are expressed as median and interquartile ranges. To compare differences between saline and the respective DEP groups, the Mann Whitney Rank Sum test was used. The Sigmastat v.9.0 program was used for the analyses. The significance level was set at 5%.

## Results

### Determination of particle size distributions in the suspension

The frequency distribution of particle diameters in saline shows that our DEP contains 90% of particles with a diameter below 25.29 μm, followed by 50% of particles below 8.96 μm and also 10% below 2.71 μm. The average sizes of the particles were 11.84 and 5.79 μm according to their volume and surface, respectively. DEP metal and organic contents of this particulate matter were previously reported.

### The effects of DEP exposure on respiratory mechanics and lung morphology

**Figure 1:**
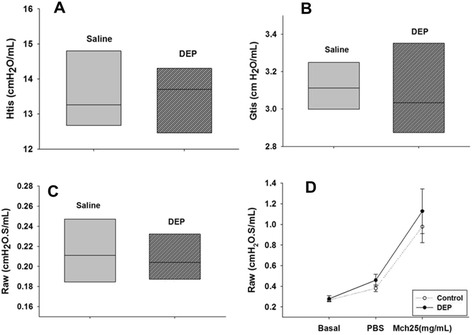
**Respiratory mechanics parameters in saline or DEP exposed mice during 90 days. (A)** tissue elastance (Htis), **(B)** tissue damping (Gtis) and **(C)** airway resistance (Raw). The central line defines the median, the borders above and below the shaded areas represent the 25% and 75% percentiles, respectively, and the error bars represent the 5% and 95% percentiles. The grey box represents saline group and stripped box represent DEP group. **(D)** Respiratory mechanics after methacholine challenge in saline (white circle) or DEP (black circle) exposed mice, where Raw means airway resistance. There were not statistically difference significant between groups.

**Figure 2:**
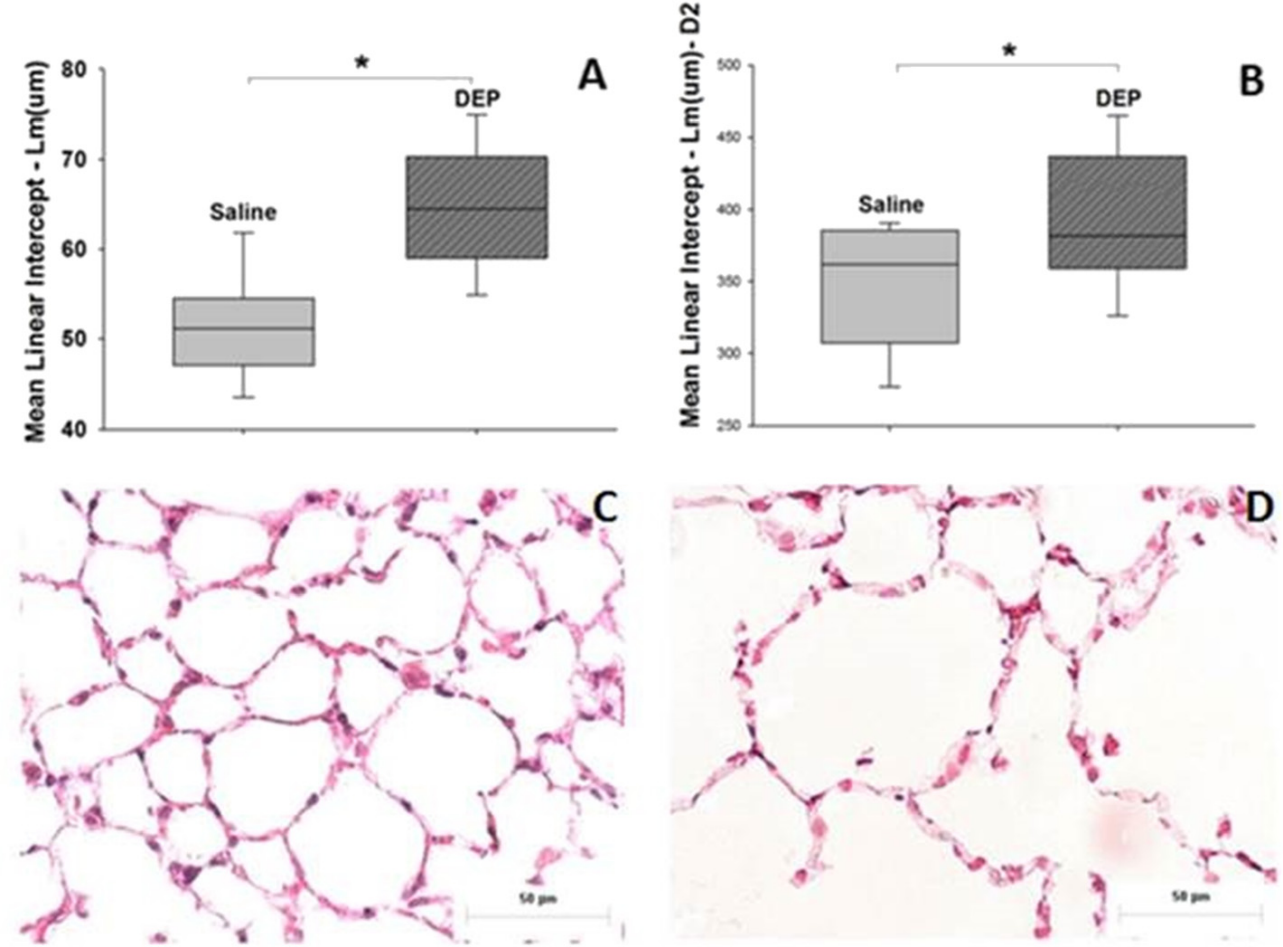
**Airspace enlargement in the lungs in saline or DEP exposed mice. (A)** Mean linear intercept (Lm) (*p ≤ 0.001) and **(B)** parameter D2 (*p = 0.038). **Lung parenchyma morphology.** Photomicrography of mice lung parenchyma (HE), 90 days after exposure with saline **(C)** or a concentration of DEP of 30 μg/10 μL **(D)**. Scale bar = 50 μm.

DEP exposure did not alter the respiratory mechanics under basal conditions and during methacholine challenge [(basal_saline_ = 0.461 ± 0.052 cmH_2_0.S/mL; PBS_saline_ = 0.578 ± 0.109 cmH_2_0.S/mL; Mch_saline_. = 1.509 ± 0.267 cmH_2_0.S/mL) and (basal_DEP_ = 0.444 ± 0.069 cmH_2_0.S/mL; PBS_DEP_ = 0.622 ± 0.181 cmH_2_0.S/mL; Mch_DEP_ = 1.663 ± 0.549 cmH_2_0.S/mL)]. Tissue elastance (Htis): saline = 13.264 cmH_2_0/mL [12.309 – 15.447] and DEP = 13.706 cmH_2_0/mL [10.800 – 15.251], damping (Gtis): saline = 3.113 cmH_2_0/mL [2.729 – 3.558] and DEP = 3.034 cmH_2_0/mL [2.729 – 3.558] and airway resistance (Raw): saline = 0.211 cmH_2_0.S/mL [1.014 – 0.267] and DEP = 0.204 cmH_2_0.S/mL [0.173 – 0.242] were not different between saline and DEP-exposed mice (Figure 1). No differences were observed in the thickness of the bronchiolar epithelium in DEP-exposed compared to saline-exposed animals (Table 1). Mean linear intercept (Lm) (p ≤ 0.001) (Figure 2A) and the parameter D2 (Figure 2B) (index based on the statistical distribution of mean airspace diameter-D0) (p = 0.038) increased in DEP-exposed animals; D0_saline_ = 110.285 [105.890 – 120.315]; D0_DEP_ = 110.430[107.811–116.989]; D2_saline_ = 361,827 [311.461 – 384.803]; D2_DEP_ =381.844 [359.245 – 430.586]. The long-term intranasal DEP exposure during 90 days damaged the lung parenchyma, which caused modifications in the distal airspaces, resulting in alveolar enlargement (Figure 2C, D).

**Table 1:** **Density of macrophages (MAC2+ cells), CD4+ T cells, CD20+ B cells, IL-13 epithelial cells, Muc5ac (mRNA), bronchial epithelial thickness in DEP and saline groups after 90 days of exposure**

	**Saline**	**DEP**
**MAC2+** *(cells/mm* ^*2*^ *)*	47.74 (0.00-143.54)	79.64 (52.36-158.73)
**CD4+** *(cells/mm* ^*2*^ *)*	210.22 ± 98.43	221.43 ± 108.89
**CD20+** (cells/mm^2^)	793.41 (664.34-893.47)	888.56 (761.59-1111.11)
**Muc5ac**	0.10 (0.04-0.27)	0.15 (0.00-0.72)
**Bronchial epithelial thickness** *(μm)*	9.20 (7.55-11.33)	6.89 (6.47-10.51)
**IL-13** *(area+/area)*	0.34 ± 0.11	0.11 ± 0.10*

Date are presented as means ± SD or medians (interquartile ranges). * p = 0.008.

**Figure 3:**
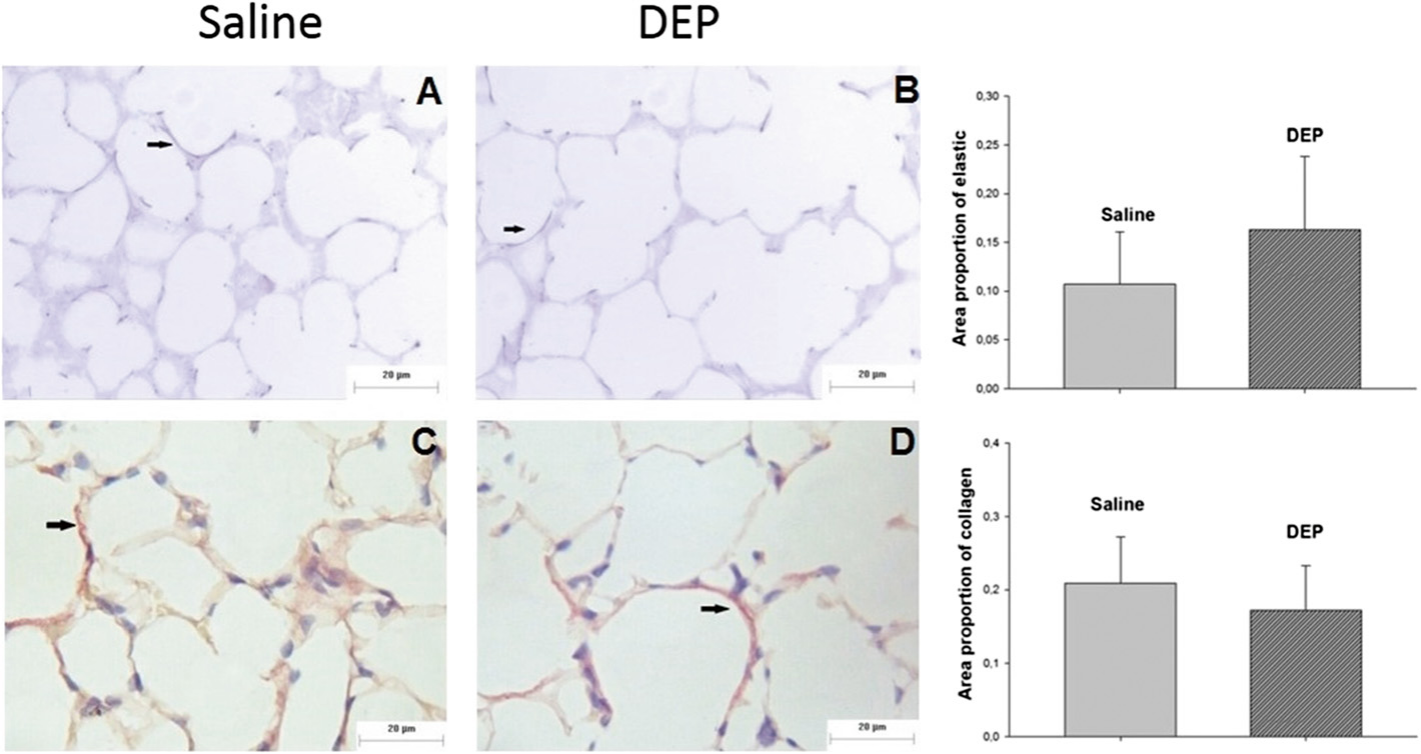
**Elastic fibers (resorcin-fuchsin) and collagen fibers (sirius red) in the parenchyma region.** Photomicrography of mice lung parenchyma showing elastic fibers **(A, B)** and collagen fibers **(C, D)** in saline **(A, C)** and DEP group **(B, D)**. There were no statistically significant differences among groups in the proportion of elastic (p = 0.08) or collagen fibers (p = 0.208) occupied in the lung parenchyma. Scale bar = 20 μm.

There were no statistically significant differences among groups in the proportion of elastic (p = 0.08) or collagen fibers (p = 0.208) occupied in the lung parenchyma (Figure 3).

### Effects of DEP exposure on lung inflammation

We examined the cellular profile of BAL fluid 24 h after the last intranasal instillation. The administration of DEP did not increase the total BAL cell counts when compared to the saline group after 90 days. Differential cell counts in the BAL showed differences in lymphocyte numbers between groups (p = 0.017) (Table 2). The expression of IL- 4, IL −10, IL-13 and INF-γ were examined in the BAL supernatants of the study groups. No statistical differences in BAL levels of IL-4, IL-10 and IL- 13 (Table 3) were noted. However, INF-γ levels decreased in the DEP-exposed group compared to the saline group (p = 0.03).

**Table 2:** **Differential cell counts in the bronchoalveolar lavage between DEP and saline groups**

**cells/mLx10** ^**4**^	**Saline**	**DEP**
**Neutrophils**	0.004 ± 0.005	0.007 ± 0.008
**Macrophages**	1.801 ± 0.546	2.900 ± 1.327
**Lymphocytes**	0.051 ± 0.060	0.235 ± 0.301*

Values are mean ± SD. * p = 0.017.

**Table 3:** **Cytokines expression in the bronchoalveolar lavage of mice treated with DEP and saline after 90 days of exposure**

**Cytokines (pg/mL)**	**Saline**	**DEP**
**IL- 4**	6.15 ± 3.70	8.89 ± 2.41
**IL-10**	476.84 ± 241.95	305.26 ± 200.04
**IL-13**	408.6 ± 328.02	318.16 ± 241.86
**INF-gamma**	131.30 (109.46-289.36)	93.22 (84.33-102.25)*

Date are presented as means ± SD or medians (interquartile ranges). IL = Interleukin, INF-γ = Interferon-gamma. * p = 0.03.

**Figure 4:**
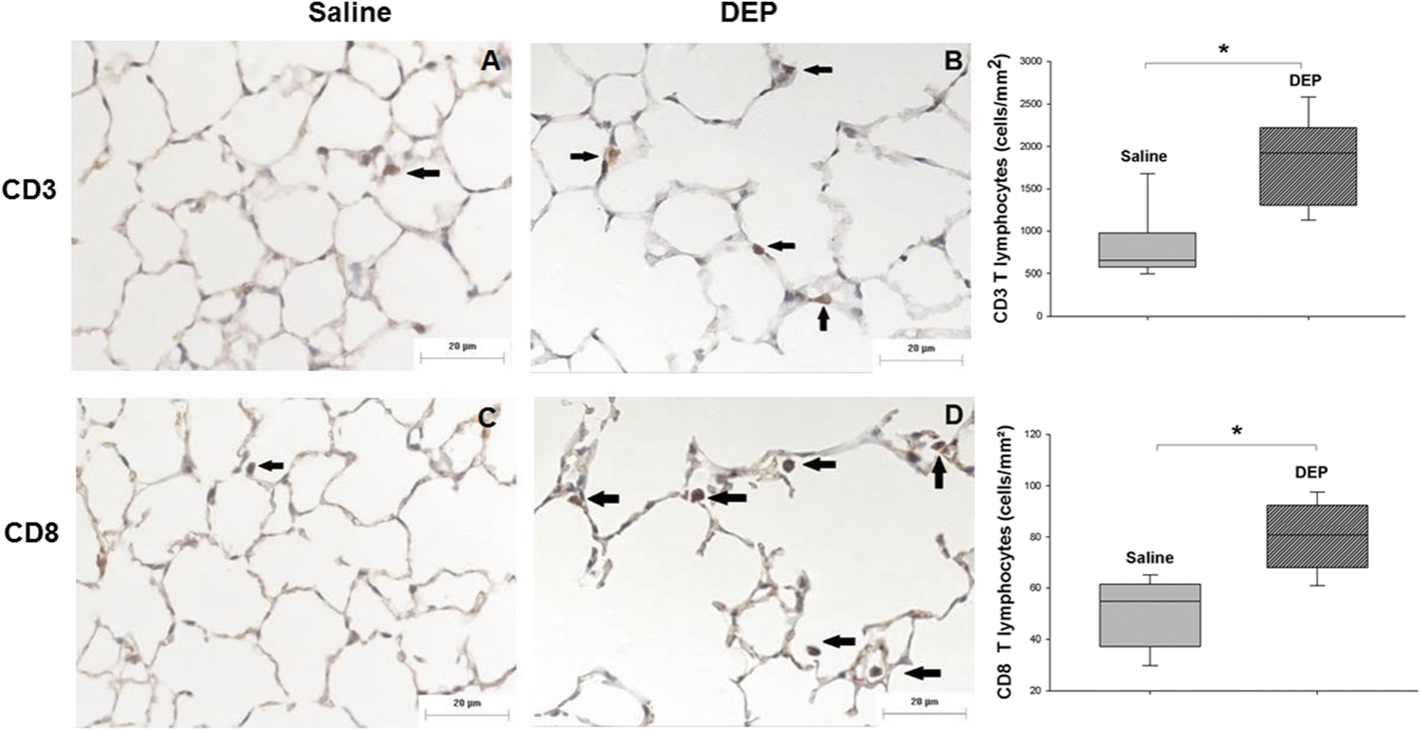
**CD3+ and CD8+ T cells density in lung parenchyma in saline or DEP exposed mice.** Photomicrography of mice lung parenchyma showing CD3+ **(A,B)** and CD8+ T cells **(C, D)** in saline **(A, C)** and DEP group **(B, D)**. Observe the increased density of these cells in the DEP group. Scale bar = 20 μm. The graphs show expression of CD3+ T lymphocytes and CD8+ T lymphocytes in saline and DEP group. The median is represented as horizontal bars. CD3+ T lymphocytes: *p ≤ 0.001 compared with saline group. CD8+ T lymphocytes: *p ≤ 0.001 compared with saline group.

In the bronchiolar epithelium, there was reduced expression of IL-13 in the DEP group when compared to the saline group (p = 0.008). In the lung parenchyma, there were no statistically significant differences in MAC2+ macrophage, CD4+ T cell and CD20+ B cell counts (Table 1) between groups. However, there was an increase in the number of CD3+ T cells (p ≤ 0.001) and CD8+ T cells (p ≤ 0.001) in the DEP group when compared with the saline group (Figure 4). The pulmonary expression of Muc5ac mRNA was not statistically different between groups (Table 1).

## Discussion

In this study we demonstrated that chronic exposure to diesel particles trigger alterations in lung structure of the alveolar parenchyma, associated with CD8+ T cell inflammation and decreased IFN-γ production, that are not associated with impaired lung function or significant extracellular matrix remodeling. Many studies showed associations between air pollution and exacerbations of pre-existing COPD, but the role of air pollution in the development and progression of COPD [9,21-25] is still uncertain. Particle retention in lung tissue results in a chronic, low-grade inflammatory response that may be pathogenetically important in the progression of lung disease. It is possible that longer exposures (in animals or in real life) could have a more significant impact on lung mechanics or remodeling, as it is observed in cigarette smoking models. In addition, it is possible that in pre-injured lungs, like the smoker’s lungs, chronic exposure to DEP/air pollution could have a synergic effect on the development of emphysema. We have previously demonstrated that exposure to ambient particles accelerates the development of protease induced emphysema in mice [10].

Environmental factors play a critical role in the regulation of the innate and adaptive immune responses that might be associated with the development of allergic or chronic obstructive pulmonary diseases. In our study, we observed decreases in type 1 (IFN-γ) without any differences in IL-10 in the BAL (Table 3). A decrease in IFN-γ production in the BAL of smokers and a downregulation of M1 genes has been previously described in smokers and COPD [26]. Interestingly, we observed a lower expression of IFN-γ in the BAL of DEP treated animals, without a concomitant increase in macrophages [27]. This finding suggests that M1/M2 balance could be also modified by DEP exposition, explaining the altered immune allergic or infectious responses described in exposed animals [28,29].

Exposure to low dose DEP over a period of 90 days caused alveolar enlargement with a CD8+ T lymphocytic inflammation without a concomitant increase in macrophage and neutrophil numbers or an increase in cytokines. Similarly, Biselli et al. [30] studied the inflammatory and structural pulmonary effects of exposure to residual oil fly ash (ROFA) in mice for 2 months. The authors could not detect increases in inflammatory cells but observed early signs of alveolar enlargement in the animals exposed to ROFA. How can we explain these findings? It is possible that other tissue injury pathways are activated during diesel exposure, such as the cytolitic cascade of granzymes/perforin. Upregulation of granzyme B in CD8+ and non-CD8+ cells has been demonstrated to be an early phenomenon of small airway wall remodelling in centrilobular emphysema in patients with COPD [31]. The identification of increased CD8+ T cells in the parenchyma confirm the findings of a previous study by Deiuliis et al. [31], that investigated the effects of chronically inhaled particulate matter <2.5 μm (PM_2.5_) on inflammatory cell populations in the lung, mediastinal lymph nodes, spleen, and circulation [32].

Another possibility is the induction of air pollution-induced autophagy in lung cells. Several studies have demonstrated that cigarette smoke induces autophagy in lung cells [33], and this autophagic process appears to play a critical role in the pathogenesis of emphysema [33-35]. Deng et al. 2013 found that PM_2.5_ can elicit oxidative stress, resulting in accumulation of intracellular reactive oxygen species (ROS) and autophagic cell death in human epithelial lung A549 cells [36].

It is possible that longer exposures would be necessary to detect more pronounced inflammatory or ECM (extracellular matrix) changes.

In our protocol, DEP route administration and particle size may have contributed to a proportionally higher dose of particles retained in the upper regions of the respiratory tract and the point is made that much of the instilled dose is likely to be retained in the upper airway, the more important point that this lack of penetration to the deep lung may account for some of the negative findings. Nevertheless, the presence of alveolar macrophages containing engulfed carbon particles indicates that particles reached the distal parts of the lungs. We could not find functional alterations in lung mechanics in our model. However, Lopes et al. [10] showed that morphometric parameters were more reliable for detecting the presence of emphysema than respiratory mechanics in a model of protease induced-emphysema. We also cannot exclude the possibility that the alveolar enlargement induced in our model was not severe enough to cause functional changes.

Evidence shows that lung blood vessels actively promote alveolar growth during development and contribute to the maintenance of alveolar structures throughout postnatal life. Preservation of vascular growth and endothelial survival promotes growth and sustains the architecture of the distal airspace [37]*.* Air pollution is known to induce endothelial dysfunction [38-40]. In our work, animals were exposed from postnatal week 8 onwards. We speculate that DEP could alter alveolar growth pathways by altering endothelial growth factors. Mauad et al. 2008 showed that chronic exposure to air pollution has been associated with adverse effects on mouse lung growth and development in early life. Whether pollution also alters the postnatal structure of the lungs should be investigated [41]*.*

A perception that COPD, including emphysema, is caused primarily by smoking has hindered opportunities for primary prevention, diagnosis, and treatment of these diseases [12]. However, limiting air pollution exposure in the general population does not depend solely on an individual’s actions, but also on public policy and exposure awareness programs. The World Health Organization and the American Thoracic Society have identified permanent reduction in lung function as an important potential outcome of air pollution exposure and have recognised that genetic factors may be important in determining such effects [12,42].

Increasing experimental and epidemiological evidence shows that ambient pollution alters structures involved in lung development. Here, we show that chronic exposure of adult mice to diesel particles can also affect lung structure in the absence of overt inflammation. Future studies should be conducted to elucidate the pathways related to alveolar damage caused by air pollution.

## Abbreviations

DEP: diesel exhaust particles
BAL: bronchoalveolar lavage
ELISA: enzyme-linked immunosorbent assay
RT-PCR: real time-polymerase chain reaction
Lm: mean linear intercept
PM: particulate matter
PM_2.5_: particulate matter ≤2.5 μm
COPD: chronic obstructive pulmonary disease
CAPPesq-FMUSP: Ethical Committee for Research of the School of Medicine, University of Sao Paulo
DEP: intranasal instillation of 30 μg DEP/ 10 μL of saline during 90 days
Raw: airway resistance
Gtis: tissue damping
Htis: tissue elastance
R: total respiratory system resistance
PBS: phosphate-buffered saline
D0: equivalent airspace diameter
D2: indexes based on the moments of diameter
